# A Metal Ion-Responsive Spiropyran-Based Fluorescent Color-Changing Hydrogel

**DOI:** 10.3390/ma18112573

**Published:** 2025-05-30

**Authors:** Yuxiu Yin, Xin Li, Ying Li, Hongyan Miao, Gang Shi

**Affiliations:** Key Laboratory of Synthetic and Biotechnology Colloids, Ministry of Education, School of Chemical and Material Engineering, Jiangnan University, Wuxi 214122, China

**Keywords:** spiropyran, hydrogel, plasmon resonance, fluorescence enhancement, metal ions responsiveness

## Abstract

The low fluorescence quantum efficiency of hydrophilic modified spiropyran in hydrogel matrices cannot be naturally improved during photoresponsive operation, which significantly limits their practical applications.In this study, a hybrid hydrogel system integrating metal plasmon resonance-enhanced fluorescence effects is designed through copolymerization of N,N′-bis(acryloyl)cystamine-modified Au nanoparticles (Au NPs), hydrophilic graft-modified spiropyran molecules, and N-isopropylacrylamide. This approach successfully achieves a spiropyran-based fluorescent hydrogel sensor with enhanced fluorescence intensity. Furthermore, an inverted pyramid-structured surface is engineered on the hydrogel using a template-assisted strategy, combining anti-reflection optical effects with plasmonic enhancement mechanisms. Molecular modification facilitated the integration of spiropyran and Au NPs into the hydrogel molecular chains, enhancing the dispersion of Au NPs within the hydrogel matrix and preventing fluorescence quenching from direct contact between Au NPs and spiropyran. Additionally, the anti-reflection effect of the hydrogel surface microstructure and the plasmon resonance effect of Au NPs were crucial in boosting the sensor’s fluorescence. Finally, the fluorescence intensity of the hydrogel increased by 10.2 times. In addition, under the action of excitation light, this sensor exhibited dual responsiveness of colorimetry and fluorescence, allowing for the sensing of heavy metal ions. The limit of detection for Zn^2+^ is as low as 0.803 μM, and the hydrogel exhibited more than 10 cycles of photo-isomerization and ion responsiveness.

## 1. Introduction

Spiropyran is a representative photochromic material exhibiting reversible structural transformation that enables reversible switching between the non-fluorescent closed-ring SP form and fluorescent open-ring MC form under stimuli including stress, light irradiation, pH variation, and temperature changes [1,2,3,4,5,6,7]. However, due to its poor water solubility, spiropyran is difficult to disperse directly into the hydrogel matrix [8,9].

In current research, two strategies have been developed to address this challenge. (1) The first method involves encapsulating spiropyran molecules within the hydrophobic core formed by polymer segments through emulsion polymerization, while the hydrophilic shell provides excellent water solubility without compromising the optical properties of spiropyran [10,11,12]. However, the thickness and opacity of the shell material will reduce the photochromic properties of the composite hydrogel. For example, Zheng et al. [13] initially utilized Tween 80 to form latex particles encapsulating spiropyran and methyl methacrylate, followed by the addition of hydrophilic acrylamide. Finally, white-light-initiated polymerization yielded a polyacrylamide-co-methyl methacrylate/spiropyran smart fluorescent hydrogel sensor capable of responding to multiple stimuli, such as light, temperature, and mechanical forces. However, such hydrogel sensors are difficult to apply in chemical sensing because water-soluble stimuli struggles to penetrate the hydrophobic core and react with spiropyran. (2) The second method involves grafting hydrophilic groups (such as carboxyl, sulfonate, and methacrylate groups) onto spiropyran molecules to impart water solubility [9,14]. These water-soluble spiropyran molecules can be used to synthesize uniform hydrogel materials, achieving a photochromic cycling stability of five to seven times. For example, Liao et al. [15] grafted water-soluble acryloyl-α-cyclodextrin onto spiropyran to enhance its hydrophilicity, followed by copolymerization with N-isopropylacrylamide to produce a photo-switchable and thermally enhanced fluorescent hydrogel sensor. However, this approach cannot prevent the energy loss caused by inelastic collisions between the MC and water molecules, which rapidly dissipate as heat into the surrounding medium. This significantly reduces the fluorescence quantum yield of MC, leading to a substantial decrease in its emissions intensity. Consequently, current spiropyran-based fluorescent hydrogels prepared through grafting modification exhibit limited sensitivity for trace metal ion detection, thereby constraining their applications in advanced sensing technologies.

While leveraging the plasmon resonance effect of metallic NPs to enhance fluorescence intensity in matrices represents a simple and efficient strategy [16], the inherent aggregation tendency of NPs during hydrogel dispersion severely compromises their ideal fluorescence enhancement performance. In this work, we present a spiropyran-based fluorescent hydrogel sensor with inverted pyramid architecture, fabricated through free radical copolymerization of carbon-carbon double-bond-functionalized Au NPs, hydrophilically modified spiropyran, and hydrogel monomers on silicon templates. The anti-reflection effect of the inverted pyramid structure and the plasmon resonance effect of the Au NPs can effectively improve the fluorescence intensity of the sensor, thereby enhancing its detection limit for heavy metal ions.

## 2. Materials and Methods

### 2.1. Materials

2,3,3-Trimethylindole, 3-iodopropionic acid, N,N′-bis(acryloyl)cystamine (DA), chloroauric acid, N,N′-methylenebisacrylamide, and 5-nitrosalicylaldehyde were purchased from Shanghai Adamas Reagent Co., Ltd. (Shanghai, China). Piperidine, 2-butanone, potassium hydroxide (KOH), isopropanol, anhydrous ethanol, tetrahydrofuran (THF), trichloromethane, and acetone were purchased from Sinopharm Chemical Reagent Co., Ltd. (Shanghai, China). Dicyclohexylcarbodiimide (DCC), N-isopropylacrylamide (NIPAAM), sodium citrate, 4-dimethylaminopyridine (DMAP), and hydroxyethyl methacrylate (HEMA) were purchased from Beijing Innochem Technology Co., Ltd. (Beijing, China). Crystalline silicon (100) wafers were purchased from Zhejiang Lijing Optoelectronics Technology Co., Ltd. (Taizhou, China).

### 2.2. Synthesis of 2-[3-(3′,3′-Dimethyl-6-nitrospiro[chromene-2,2′-indole]-1′-yl)propanoyloxy] Ethyl 2-Methylprop-2-enoate (SPMA)

**Scheme 1:**
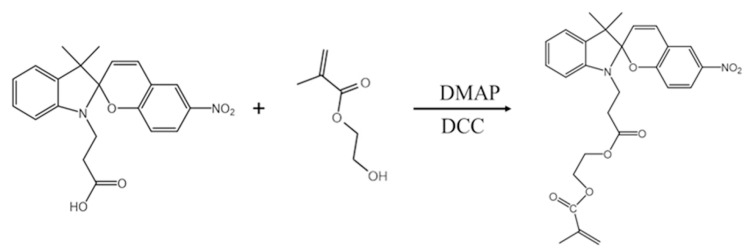
Schematic diagram of the synthesis route of SPMA.

**Figure 1:**
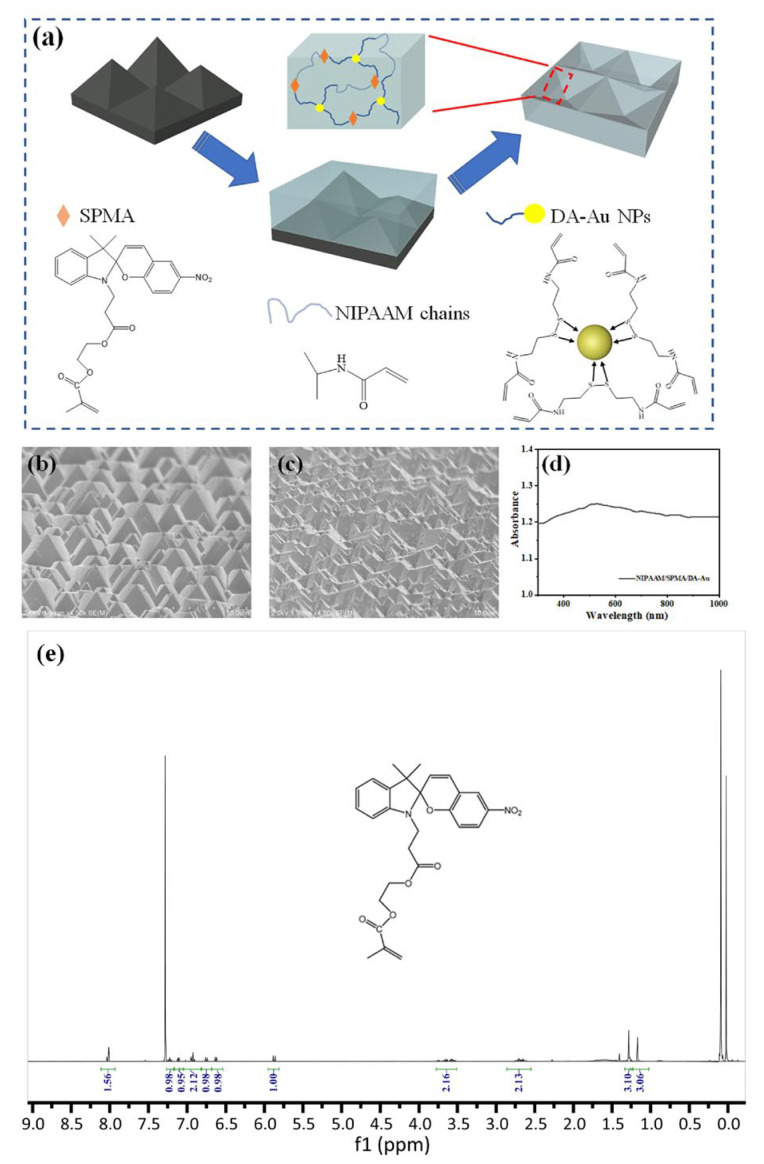
(**a**) A schematic diagram of the preparation of NIPAAM/SPMA/DA-Au hydrogel. SEM images of (**b**) the Si template and (**c**) NIPAAM/SPMA/DA-Au. (**d**) Absorption spectra of NIPAAM/SPMA/DA-Au. (**e**) 1H NMR spectrum of SPMA (400 MHz, CDCl_3_).

First, 1-(2-carboxyethyl)-3,3-dimethylindoline-6′-nitrobenzospiropyran was synthesized by referring to the previous work of our research group [17]. 1-(2-carboxyethyl)-3,3-dimethylindoline-6′-nitrobenzospiropyran (0.38 g, 1 mmol), DMAP (0.0244 g, 0.2 mmol), and HEMA (0.13 g, 1 mmol) were added to 15 mL of THF in an ice–water bath to form a mixed solution. Then, DCC (0.206 g, 1 mmol) was dissolved in 5 mL THF and slowly added dropwise into the mixed solution at 0 °C and stirred for 2 h. Subsequently, the temperature was raised to 25 °C, and the solution was stirred for 12 h. Finally, the SPMA (See Scheme 1) was obtained by filtering and washing with acetone three times. ^1^H NMR (400 MHz, CDCl_3_, ppm) δ: 1.17 (s, 3.06, 3H), 1.28 (s, 3.10, 3H); olefinic protons: 2.69 (m, 2.13, 2H), 3.62 (m, 2.16, 2H), 4.25 (d, 1.39, 1H),5.6-6.1 (d, 2.73.00, 3H), 6.61 (d, 0.98, 1H), 6.75 (d, 0.98, 1H); and aromatic protons: 6.93 (dd, 2.12, 2H), 7.18 (d, 0.95, 1H), 7.23 (td, 0.98, 1H), 8.02 (m, 1.56, 2H) (Figure 1e).

### 2.3. Synthesis of Gold Nanoparticles Modified by DA (DA-Au)

First, 20 mL of chloroauric acid (0.01 wt%) was added to a round-bottomed flask and boiled under stirring and reflux. Then, 0.14 mL of sodium citrate (1 wt%) was quickly added to the solution and stirred for 30 min to obtain a gold nanoparticle (Au NP) solution [18]. Finally, 10 mL of DA (0.01 wt%) was mixed with the Au NP solution to obtain DA-Au.

### 2.4. Synthesis of NIPAAM/SPMA/DA-Au Hydrogel

First, a silicon template with micro-pyramid structures was obtained by etching in KOH solution for 30 min [19,20]. Then, 2 g of NIPAAM, 0.02 g of SPMA, 0.02 g of N,N′-methylenebisacrylamide, 0.02 g of ammonium persulfate, and 1 mL of DA-Au solution were added to 10 mL of water and mixed evenly. The mixture was poured onto the surface of a silicon template and allowed to stand at 60 °C for 3 h to form a hydrogel. Finally, the hydrogel was peeled off from the surface of the silicon template to obtain NIPAAM/SPMA/DA-Au hydrogel with inverted pyramid structures.

### 2.5. Characterization

The surface morphology of the sample was characterized by field emission scanning electron microscopy (FESEM, Hitachi S-4800, Hitachi High-Tech Corporation, Tokyo, Japan). The molecular structure was analyzed by ^1^H NMR (AVANCE III HD-400, Brugg Group, Bruker, Switzerland). Absorption and transmission spectra were obtained using a Shimadzu 3600 UV-Vis-NIR spectrophotometer (Shimadzu Corporation, Kyoto, Japan). Unless otherwise specified, visible light irradiation was realized by a Xenon lamp (MC-PF300C, Beijing Merry Change Technology Co., Ltd., Beijing, China) equipped with a 400 nm cut-off filter, and the irradiation time was 3 min. Ultraviolet light irradiation was realized by a mercury lamp (MC-M500, Beijing Merry Change Technology Co., Ltd., Beijing, China) equipped with a 365 nm filter, and the irradiation time was 120 s. Photoluminescence analysis was performed with a fluorescence spectrometer (FS5, Edinburgh Instruments Ltd., Edinburgh, UK). Fluorescence lifetime analysis was conducted using an ultrafast time-resolved fluorescence lifetime spectrometer (Lifespec II, Edinburgh Instruments Ltd., Edinburgh, UK).

## 3. Results and Discussion

### 3.1. Fabrication of NIPAAM/SPMA/DA-Au

The preparation process of the NIPAAM/SPMA/DA-Au fluorescent hydrogel sensor is depicted in Figure 1a. The NMR spectrum analysis of SPMA confirmed successful esterification of spiropyran with methacrylic anhydride. (Figure 1e). Owing to differential etching kinetics in KOH solution, the silicon (111) plane exhibits significantly reduced etch rates compared to the (100) plane [19]. Utilizing this anisotropic wet etching, a micro-pyramid structure was constructed on the surface of single-crystal silicon to obtain a silicon template. The average height of the micro-pyramids on the silicon template surface was 5 μm, with the angle between the side face and the plane being approximately 54.7°, as depicted in Figure 1b. Subsequently, the silicon template underwent hydrophobic treatment to ensure that the hydrogel could be smoothly peeled off from its surface. Meanwhile, a layer of DA molecules was modified on the surface of Au NPs through the reaction between thiol groups and Au to obtain DA-Au, while spiropyran was functionalized with hydrophilic HEMA through esterification to produce SPMA. Then, the mixed solution containing NIPAAM, SPMA, and DA-Au was poured onto the surface of the silicon template and subjected to thermal polymerization to obtain the hydrogel. Finally, the hydrogel was separated from the silicon template, yielding a fluorescent hydrogel sensor with an inverted pyramid surface structure designated as NIPAAM/SPMA/DA-Au, as shown in Figure 1c. This inverted pyramid structure serves as an effective anti-reflection layer, enhancing incident photon capture [21]. Figure 1d shows the absorption spectrum of NIPAAM/SPMA/DA-Au without UV treatment. NIPAAM/SPMA/DA-Au exhibits a distinct absorption peak at 525 nm, which is attributable to the plasmon resonance absorption of Au NPs, indicating the successful doping of Au NPs within the hydrogel.

### 3.2. Fluorescence and Photochromism Performance

**Figure 2:**
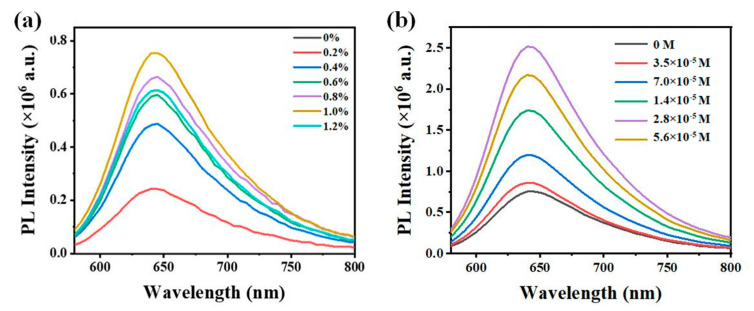
Fluorescence spectra of NIPAAM/SPMA/DA−Au after ultraviolet irradiation for 2 min with (**a**) different SPMA contents and different DA−Au NPs contents. (**b**) different DA−Au NPs contents.

The content of SPMA directly affects the grafting content of spiropyran within the NIPAAM/SPMA/DA-Au hydrogel substrate, critically determining its fluorescence characteristics. As can be seen from the fluorescence spectra in Figure 2a, after the substrate was treated with ultraviolet light, a strong fluorescence emission peak appeared at 645 nm, and the fluorescence intensity of the substrate increased with the improvement in the SPMA content. When the SPMA content reached 1%, the fluorescence intensity of the substrate reached a maximum value. Subsequently, as the SPMA content continued to increase, the fluorescence intensity of the substrate began to decrease. This was due to the fact that an excessively high content of SPMA led to the aggregation of MC, resulting in self-fluorescence quenching. The content of Au NPs also significantly affected the fluorescence performance of the hydrogel substrate, as shown in Figure 2b. Compared with the substrate without Au NPs, the fluorescence intensity of the substrate with Au NPs significantly improved, indicating that the plasmonic resonance effect of Au NPs within the substrate enhances the fluorescence emission of spiropyran [22]. As the concentration of Au NPs increases, the fluorescence intensity of the substrate also increases. When the concentration of Au NPs is 2.8 × 10^−4^ M, the fluorescence intensity of the substrate reaches a maximum. However, an excessively high concentration of Au NPs causes a decrease in the fluorescence intensity of the substrate. This is because a high concentration of Au NPs increases the chance of contact with spiropyran, leading to non-radiative energy transfer and quenching the fluorescence of spiropyran.

**Figure 3:**
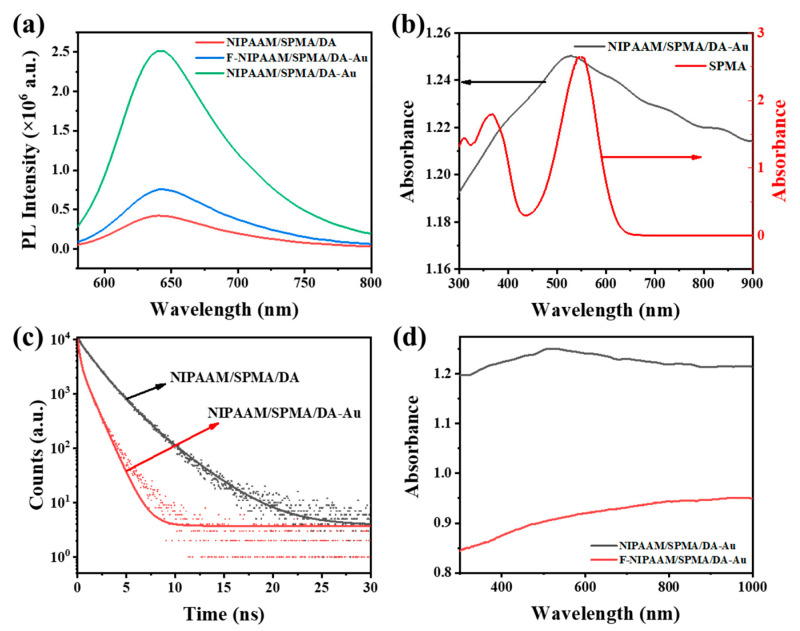
(**a**) Fluorescence spectra of F-NIPAAM/SPMA/DA-Au, NIPAAM/SPMA/DA-Au, and NIPAAM/SPMA/DA after ultraviolet irradiation for 2 min. (**b**) Absorption spectra of NIPAAM/SPMA/DA-Au (black) and SPMA after ultraviolet irradiation for 2 min (red). (**c**) Time-resolved transient photoluminescence spectra of NIPAAM/SPMA/DA (black) and NIPAAM/SPMA/DA-Au (red) after ultraviolet irradiation for 2 min. (**d**) Absorption spectra of NIPAAM/SPMA/DA-Au (black) and F- NIPAAM/SPMA/DA-Au (red).

The NIPAAM/SPMA/DA-Au hydrogel exhibits substantially enhanced fluorescence intensity relative to both planar (F-NIPAAM/SPMA/DA-Au) and gold-free (NIPAAM/SPMA/DA) counterparts, as shown in Figure 3a. It is mainly attributed to three aspects. (1) Resonance Energy Transfer: The plasmonic resonance absorption peak of Au NPs overlaps significantly with the spiropyran absorption peak, as shown in Figure 3b. This results in the far-field scattering energy of Au NPs being transferred to spiropyran via resonance energy transfer, thereby greatly enhancing the excitation efficiency and radiation rate of spiropyran and increasing the fluorescence intensity of the spiropyran. (2) Local Field Enhancement: The plasmonic resonance absorption peak of Au NPs is close to the excitation wavelength (540 nm), which generates an enhanced local electromagnetic field. This significantly increases the probability of radiative transitions of electrons within the spiropyran, providing more rapid decay channels and thereby reducing the fluorescence lifetime and enhancing fluorescence emissions. As shown in Figure 3c, the fluorescence lifetime of NIPAAM/SPMA/DA-Au is significantly lower than that of NIPAAM/SPMA/DA. (3) Anti-Reflective Structuring: The inverted pyramid structure can effectively slow down the refractive index mutation of air to the substrate, reduce the reflection of the excitation light and, thus, improve the absorption of the excitation light, which can enhance fluorescence emissions. As shown in Figure 3d, the NIPAAM/SPMA/DA-Au hydrogel substrate with an inverted pyramid structure has a significantly higher absorption efficiency for incident light than F-NIPAAM/SPMA/DA-Au.

**Figure 4:**
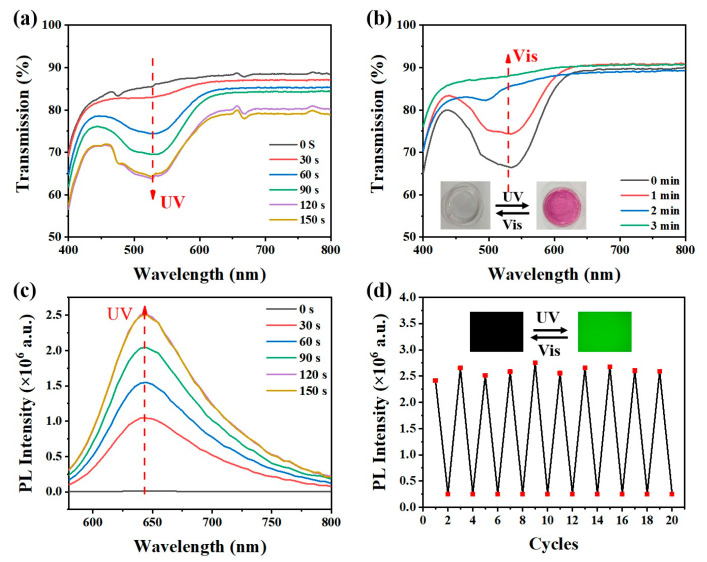
(**a**) Transmission spectra of NIPAAM/SPMA/DA-Au with different times under ultraviolet irradiation. (**b**) Transmission spectra of discolored NIPAAM/SPMA/DA-Au with different times under visible irradiation (inset: photos of NIPAAM/SPMA/DA-Au with color changes). (**c**) Fluorescence spectra of NIPAAM/SPMA/DA-Au with different times under ultraviolet irradiation. (**d**) The cyclic fluorescence intensity at 645 nm of NIPAAM/SPMA/DA-Au under ultraviolet and visible irradiation (inset: fluorescence switching photos under a fluorescence microscope).

The NIPAAM/SPMA/DA-Au hydrogel substrate exhibits excellent photoresponsive properties. As shown in Figure 4a, under UV light irradiation, the transmittance of the substrate at 535 nm gradually decreases and changes from colorless to red, which is attributed to the gradual isomerization of spiropyran from SP (colorless) to MC (red). When the ultraviolet irradiation time is extended to 120 s, the transmittance of the NIPAAM/SPMA/DA-Au at 535 nm reaches the lowest value, indicating that all the spiropyran has been converted to MC. As shown in Figure 4b, under visible light irradiation, the transmittance of NIPAAM/SPMA/DA-Au at 535 nm gradually increases and gradually changes from red to colorless because spiropyran gradually isomerizes back to SP. The MC has a distinct fluorescence peak at 645 nm. Under ultraviolet light irradiation, as the content of MC increases, the fluorescence of the hydrogel gradually enhances, as shown in Figure 4c. Consistent with the transmission spectrum, the fluorescence intensity of NIPAAM/SPMA/DA-Au reaches a maximum value when exposed to ultraviolet light for 120 s. In addition, NIPAAM/SPMA/DA-Au also exhibits good fatigue resistance, with no significant fluorescence intensity attenuation appearing within 10 cycles, as shown in Figure 4d.

### 3.3. Metal Ion Responsiveness

**Figure 5:**
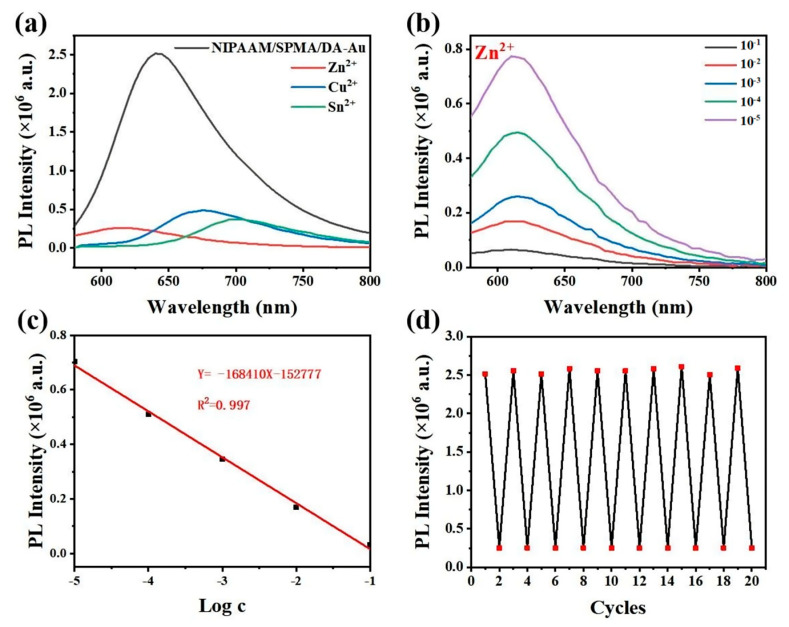
(**a**) Fluorescence spectra of NIPAAM/SPMA/DA−Au with different metal ions (10^−3^ M in water). (**b**) Fluorescence spectra of NIPAAM/SPMA/DA-Au when intruding into aqueous solutions of different concentrations of Zn^2+^ ions for 30 s after ultraviolet irradiation for 2 min. (**c**) Linear plots of fluorescence intensity versus the logarithm of Zn^2+^ concentration. (**d**) Cyclic fluorescence intensity measurements of NIPAAM/SPMA/DA−Au with Zn^2+^.

The negatively charged phenolic hydroxyl group of MC can form a complex with heavy metal ions. This process is accompanied by the electron rearrangement of MC, resulting in a shift in the MC fluorescence peak position and fluorescence quenching [23,24]. Consequently, the NIPAAM/SPMA/DA-Au hydrogel substrate can be utilized to ascertain the responsiveness of heavy metal ions in aqueous solutions. After treating the substrate with ultraviolet light, it was submerged in aqueous solutions containing 10^−3^ M Zn^2+^, Sn^2+^, and Cu^2+^, respectively. The corresponding MC fluorescence peak shifted from 645 nm to 615 nm, 665 nm, and 690 nm, and the fluorescence intensity was clearly quenched, as shown in Figure 5a. Furthermore, the fluorescence intensity of NIPAAM/SPMA/DA-Au exhibited a linear relationship with the concentration of metal ions. As illustrated in Figure 5b,c, the fluorescence intensity of NIPAAM/SPMA/DA-Au continuously decreased with the increase in Zn^2+^ concentration, and the substrate fluorescence intensity had a linear relationship with the Zn^2+^ concentration. The linear analysis equation is *Y* = −168410*X* − 152777 (with concentrations ranging from 10^−1^ M to 10^−5^ M). The limit of detection (LOD) for Zn^2+^ is 0.803 μM (LOD = 3*S*_b_/*S*, where *S*_b_ = 0.04516), indicating that the hydrogel substrates have the potential to detect Zn^2+^. Additionally, metal ions can be released when MC is isomerized back to SP under visible irradiation, so the hydrogel substrate can be reused. As demonstrated in Figure 5d, the fluorescence intensity of the NIPAAM/SPMA/DA-Au hydrogel substrate is almost not attenuated over 10 cycles of Zn^2+^ responsiveness, proving that it has excellent fatigue resistance in the field of metal ion responsiveness.

## 4. Conclusions

In summary, this study demonstrates the synthesis of a spiropyran-integrated fluorescent hydrogel sensor (NIPAAM/SPMA/DA-Au) via copolymerization of N-isopropylacrylamide (NIPAAM), hydrophilically functionalized spiropyran methacrylate (SPMA), and dopamine-grafted gold nanoparticles (DA-Au NPs). The fluorescence amplification arises from the synergistic coupling between the localized surface plasmon resonance (LSPR) of Au NPs embedded in the hydrogel matrix and the broadband antireflection enabled by the surface-architected inverted pyramidal topography. Critically, the sensor achieves exceptional sensitivity toward divalent metal ions (Zn^2+^, Sn^2+^, Cu^2+^), with a remarkably low detection limit of 0.803 μM for Zn^2+^. Moreover, it retains its initial sensitivity across six consecutive detection cycles, confirming exceptional fatigue resistance for repetitive ion monitoring applications. 

## Data Availability

The original contributions presented in this study are included in the article. Further inquiries can be directed to the corresponding authors.
